# Key CMM Combinations in Prescriptions for Treating Mastitis and Working Mechanism Analysis Based on Network Pharmacology

**DOI:** 10.1155/2019/8245071

**Published:** 2019-02-19

**Authors:** Diyao Wu, Xinyou Zhang, Liping Liu, Yongkun Guo

**Affiliations:** ^1^School of Pharmacy, Jiangxi University of Traditional Chinese Medicine, Nanchang, Jiangxi Province 330004, China; ^2^College of Computer Science, Jiangxi University of Traditional Chinese Medicine, Nanchang, Jiangxi Province 330004, China

## Abstract

**Aims:**

Using both data mining and network pharmacology methods, this paper aims to construct a molecule-target-disease network for medicines used for treating mastitis, mine out targets, and signaling pathways related to mastitis and explore the mechanism of Chinese materia medica (CMM) prescriptions in treating mastitis.

**Methods:**

A total of 131 CMM prescriptions for treating mastitis were collected from clinical practice and related literatures. A database of prescriptions for treating mastitis (DPTM) was then constructed. Based on data mining method, Traditional Chinese Medicine Inheritance Support System (TCMISS) was employed to mine out high-frequency CMM and key CMM combinations in DPTM. Subsequently, TCM Systems Pharmacology Database and Analysis Platform (TCMSP) and Traditional Chinese Medicine Information Database (TCM-ID) were searched for the targets of ingredients of high-frequency CMM. Then, Bioinformatics Analysis Tool for Molecular Mechanism of TCM (BATMAN-TCM) was searched for diseases and signaling pathways corresponding to the targets of key CMM combinations. The obtained results were denoted as results 1. In addition, human disease database MalaCards was searched for targets and signaling pathways related to mastitis. The obtained results were denoted as results 2. Results 1 and 2 were compared to obtain targets and signaling pathways included in both results, namely, mastitis-related targets of TCMs and mastitis-related signaling pathways that CMM involves in. Then, the biological functions of these targets and signaling pathways were investigated, on which basis the mechanism of CMM prescriptions in treating mastitis was explored.

**Results:**

A total of 12 key TCM combinations were identified. Taraxaci Herba, Glycyrrhizae Radix et Rhizoma, Paeoniae Radix Alba, semen citri reticulatae,* etc.* were CMM with the highest frequency of use for treating mastitis. The potential targets of these high-frequency CMM in treating mastitis were intercellular adhesion molecule 1 (ICAM-1), interleukin-6 (IL-6), lipopolysaccharide binding protein (LBP), and lactotransferrin. The potential signaling pathways that key CMM combinations may involve in during mastitis treatment were NF-*κ*B signaling pathway, immune system, PI3K/Akt signaling pathway, and TNF signaling pathway.

**Conclusions:**

From a perspective of network pharmacology, molecule-target-disease analysis may serve as an entry point for the research of mechanism of CMM. On this basis, we studied the mechanism of CMM prescriptions in treating mastitis by data mining and comparison of results. Our work thus provides a new idea and method for studying the working mechanism of CMM prescriptions.

## 1. Introduction

 Mastitis is a disease commonly occurring in lactating women. The most frequent type of mastitis is acute suppurative mastitis with clinical symptoms of lumps in breast, swelling, pain, fever, and pus oozing out. In traditional Chinese medicine (TCM), acute mastitis is called “breast carbuncle”. This name was first seen in the book* Zhenjiu Jiayi Jing* (women's miscellaneous disease ten, volume ten) written by Huangfu Mi of the Jin Dynasty [1]. Since then, this disease has been studied by TCM doctors of successive dynasties. Therefore mastitis has been identified in ancient times in TCM history. Rich experience has been accumulated for its treatment and many classic prescriptions have remained in use until today. However, due to the difference in clinical experience among TCM doctors and the complexity of TCM, the prescriptions for treating mastitis vary greatly from each other. Moreover, related research mainly focuses on the causes of mastitis and the summary of experience. These are a lack of in-depth research on medication rules in prescriptions and their working mechanism. In this paper, we collected prescriptions for treating mastitis from clinical research literatures and clinical practice in recent decade. The key Chinese materia medica (CMM) combinations in the prescriptions for treating mastitis as well as their potential targets and signaling pathways were analyzed. The results may provide useful information for the treatment of mastitis and the study of working mechanism of CMMs.

For the compatibility of medicines in CMM prescriptions, a “monarch-minister-assistant-messenger” rule should be followed. Various CMMs are used in combination to treating imbalance and disorders in the body. This is because the use of single CMM can hardly achieve high therapeutic efficacy, which indeed illustrates the idea of “multicomponents, multitargets, and systematic regulation” in TCM theory. Previous researches mainly attempt to explain the pharmacology of CMMs on the basis of the drug activity of single molecule and the effect of single target, which often fail to completely explain the working mechanism of CMMs.

With the introduction of systems biology and the application of bioinformatics, network pharmacology is also proposed. Based on the interaction among diseases, genes, targets, and medicines, network pharmacology enables to comprehensively investigate the effects of medicines on diseases. If key CMM combinations (namely, high-frequency CMM combinations) for treating mastitis are mined out, then a “key CMM combination-target-disease” network can be constructed. Subsequently, signaling pathway enrichment analysis of targets can be performed. Then, the mechanism of multiple compounds in the cooperative treatment of mastitis can be explained from the perspective of network pharmacology. This method agrees with the idea of holistic medicine and intuitively illustrates the mechanism of multisystem regulation in TCM. It also constructs a bridge between traditional Chinese medicine and western medicine since it enables investigating CMM prescriptions from a perspective of target-disease relationship, which is highlighted in western medicine.

## 2. Methods and Search Tools

**Figure 1:**
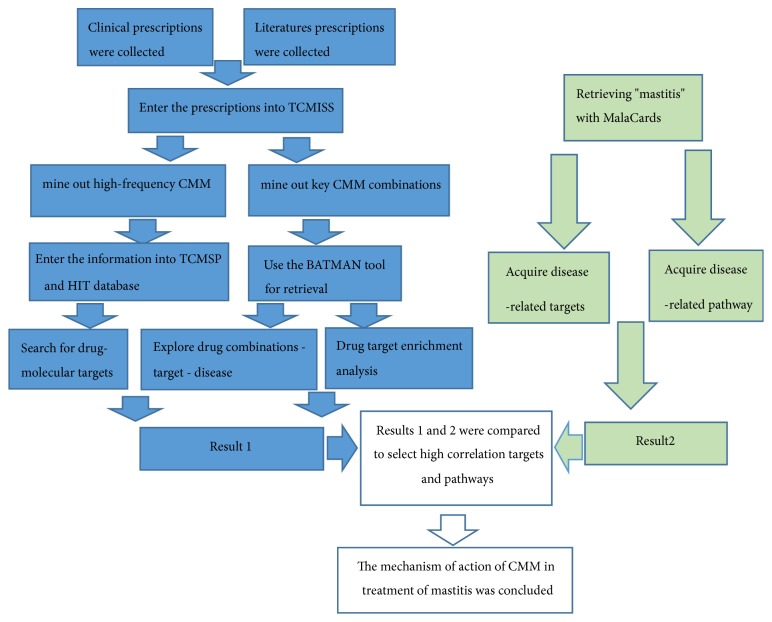
The flow chart of mining Key CMM combinations in prescriptions for treating mastitis and analyzing working mechanism based on network pharmacology.

TCM prescriptions for treating mastitis were collected from clinical practice and related literatures and then a database of prescriptions for treating mastitis (DPTM) was constructed. On the basis of data mining method, Traditional Chinese Medicine Inheritance Support System (TCMISS) was employed to mine high-frequency CMMs and key CMM combinations in DPTM. Then, Traditional Chinese Medicine Systems Pharmacology Database and Analysis Platform (TCMSP) and Traditional Chinese Medicine Information Database (TCM-ID) were searched for the targets of high-frequency CMM. Later, Bioinformatics Analysis Tool for Molecular Mechanism of TCM (BATMAN-TCM) was searched for diseases and signaling pathways corresponding to the targets of key CMM combinations. The obtained results were denoted as results 1. Furthermore, human disease database MalaCards was searched for the targets and signaling pathways related to mastitis. The obtained results were denoted as results 2. Results 1 and 2 were compared to obtain the targets and signaling pathways included in both results, namely, mastitis-related targets of CMM and mastitis-related signaling pathways that CMM involve in. Then, the biological functions of these targets and signaling pathways were identified, on which basis the mechanism of CMM prescriptions in treating mastitis was explored. A flow chart is shown in Figure 1.

### 2.1. Data Collection

DPTM consists of two kinds of prescriptions: prescriptions used in clinical practice for treating mastitis and prescriptions from related literatures for treating mastitis. First, from January 2018 to May 2018, all prescriptions for treating diseases in both outpatients and inpatients in a provincial-level breast specialist hospital were collected. Among them, prescriptions for treating mastitis were screened out. After the same prescriptions were excluded, a total of 98 prescriptions for treating mastitis were collected. Second, we searched for papers in PubMed (http://www.ncbi.nlm.nih.gov) and CNKI (http://cnki.net/) with “mastitis” and “Chinese materia medica” as two keywords. Then, a total of 45 prescriptions for treating mastitis were collected from the papers. The above prescriptions were combined and after the same prescriptions were excluded, a total of 131 prescriptions were collected, on which basis DPTM was constructed.

### 2.2. TCMISS

TCMISS is a platform focusing on analysis of CMM data, which integrates general statistics, text mining, association rules, and complex system entropy clustering method. It has already been widely applied to prescription compatibility investigation and prescription analysis [2, 3]. Prescriptions from DPTM were input one by one into TCMISS. Then, the frequencies of CMM were statistically analyzed and CMM were ordered according to their frequencies. Subsequently, association rules method was used to mine out high-frequency combinations of CMM to obtain the key CMM combinations.

### 2.3. MalaCards

MalaCards is an integrated database of human maladies and their annotations. It is modeled on the architecture and richness of the popular GeneCards database of human genes [4]. MalaCards was searched with “mastitis” as the keyword and then genes, signaling pathways, and other pieces of information related to mastitis were shown.

### 2.4. TCMSP and TCM-ID

TCMSP includes 499 CMM described in the* Pharmacopoeia of the People's Republic of China*. It involves 29,384 ingredients, 3311 targets, 837 associated diseases, and pharmacokinetic characteristics of CMM. This platform allows users to check and analyze the drug molecule-target network and drug-target-disease network, which can help reveal the working mechanism of CMM [5]. The obtained 11 CMM with the highest use frequency were input one by one in TCMSP and with additional information from TCM-ID, the ingredients of high-frequency CMM and their targets were obtained.

### 2.5. BATMAN-TCM

BATMAN-TCM is a Bioinformatics Analysis Tool for Molecular Mechanism of Traditional Chinese Medicine and is the first online bioinformatics analysis tool specially designed for the study of molecular mechanism of TCM. It mainly performs TCM ingredients' target prediction and the subsequent network pharmacology analyses of the potential targets, aiming to improve the understanding of the “multicomponent, multitargets, and multipathway” combinational therapeutic mechanism of CMM. For each ingredient of CMM, BATMAN-TCM ranks its predicted candidate targets according to the order of decreasing score given by the target prediction algorithm for the drug-target interaction prediction. It uses a similarity-based method to predict the potential targets of CMM ingredients. The core idea of this method is to rank potential drug-target interactions based on their similarity to the known drug-target interactions. If the score of a candidate target ≥ “score cutoff”, then this target will be taken as the potential target of the ingredient investigated [6].

In BATMAN-TCM, herb list was selected and the 12 key CMM combinations mined out from DPTM were input into the platform. Score cutoff was set at 80 and adjusted P-value was set at 0.05. BATMAN-TCM first predicted the potential targets of each ingredient of CMM investigated and then performed functional analyses of these targets including Gene Ontology (GO), KEGG pathway, and OMIM/TTD disease enrichment analyses. CMM ingredient-target-pathway/disease association network and biological pathways in which CMM's targets are significantly enriched were also shown.

### 2.6. Target and Signaling Pathway Screening

**Figure 2:**
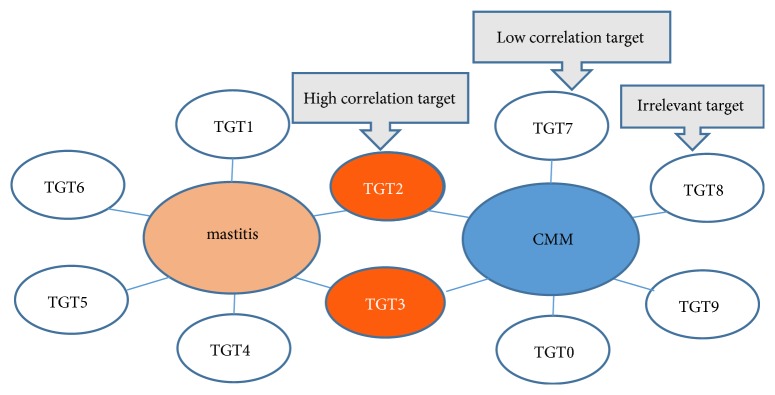
Screening of high related targets of CMM. Annotation: in this research, the orange ovals are high correlation targets, whereas the white ovals may be either low correlation targets or irrelevant targets.

**Figure 3:**
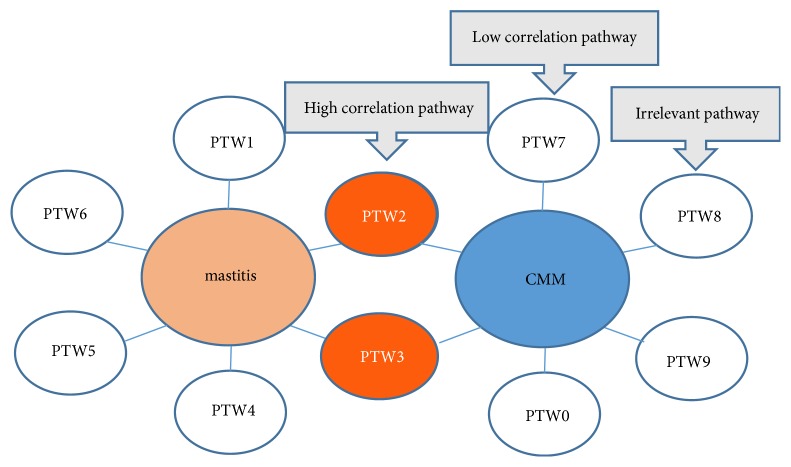
Screening of high related pathways of CMM. Annotation: in this research, the orange ovals are high correlation pathways, whereas the white ovals may be either low correlation pathways or irrelevant pathways.

The composition of CMM is very complicated. The number of targets of CMM ingredients and the number of signaling pathways that they involve in are very large. In order to screen out highly associated targets and signaling pathways, their association with mastitis should be considered. In this paper, MalaCards was searched for targets and signaling pathways related to mastitis. If CMM can also act on the same targets or signaling pathways, then these targets or signaling pathways are taken as highly associated targets and signaling pathways. In this way, the slightly relevant and irrelevant targets and signaling pathways can be excluded (Figures 2 and 3). For example, the activation of intercellular adhesion molecule 1 (ICAM-1) is highly related to mastitis, and heartleaf houttuynia herb can also act on ICAM-1. Then, ICAM-1 is considered as a highly associated target. Therefore, the potential working mechanism of herba houttuyniae may be that it inhibits ICAM-1 and thereby exerts an anti-inflammatory effect.

## 3. Results

### 3.1. Results of DPTM Mining by TCMISS

#### 3.1.1. Frequencies of CMMs

TCMISS was employed to statistically analyze the frequencies of all CMM in DPTM. DPTM includes 131 CMMs and their frequencies are shown in Table 1.

#### 3.1.2. Association Rules Mining Results

Association rules mining of DPTM was performed when support was set to ≥ 26 and confidence was set to ≥ 0.9 in TCMISS. The aim of association rules mining was to find frequent item sets, namely, key CMM combinations frequently appearing in the datasets. After repeated items were removed, 12 key CMM combinations for treating mastitis were obtained (Table 2).

### 3.2. Results of Searching in MalaCards

#### 3.2.1. Gene Targets Related to Mastitis

MalaCards was searched with “mastitis” as the keyword for targets and signaling pathways related to mastitis. The results are shown in Tables 3 and 4.

### 3.3. Results of Searching in TCMSP and HIT

After the 11 CMMs with the highest frequencies were input one by one into TCMSP and HIT (it is used to supplement the information of CMM which is unrecorded in TCMSP), the ingredients and their targets of 11 CMMs were obtained. The obtained targets were compared with those related to mastitis found in MalaCards. The same targets were screened out and taken as the mastitis-associated targets of CMM for treating mastitis (Table 5). Notably, the targets of Taraxaci Herba, semen citri reticulatae, and Semen Coicis are all not related to mastitis.

#### 3.3.1. Intercellular Adhesion Molecule 1 (ICAM-1)

ICAM-1 is a kind of membrane glycoprotein that participates in the interaction between cells or between cells and extracellular matrices [7]. During the development of inflammation, ICAM-1 has important effects on the directed migration of neutrophils and lymphocytes and their infiltration into surrounding tissues [8]. It is thus closely related to the development of inflammation. pericarpium citri reticulatae viride, Salvia miltiorrhiza Bunge, Paeoniae Radix Alba, and herba houttuyniae can all act on ICAM-1. The mechanism of them in treating mastitis might be that they inhibit ICAM-1 and thereby exert certain anti-inflammatory effects.

#### 3.3.2. Interleukin-6 (IL-6)

Interleukin (IL) is a kind of cytokine that is secreted by certain cells and has an effect on other cells. IL plays an important role in information transfer, activation and regulation of immune cells, activation, proliferation and differentiation of T and B cells, as well as inflammatory response. IL-6, as a member of interleukin family, mainly plays a role in the proliferation of B cells and antibody secretion, proliferation of T cells and CTL activation, formation of acute phase proteins by liver cells, inflammatory response,* etc.* [9]. The mechanism of Paeoniae Radix Alba, Glycyrrhizae Radix et Rhizoma, Bupleuri Radix, Semen Vaccariae, herba houttuyniae, and pericarpium trichosanthis kirilowii et multilobae in treating mastitis might be that they can decrease IL-6 level.

#### 3.3.3. Lipopolysaccharide Binding Protein (LBP)

LBP is a kind of glycoprotein existing in human and animal serum. LBP has a high affinity with lipoid A in lipopolysaccharide (LPS). It can function as a LPS carrier protein, catalyze the binding of LPS to CD14, stimulate monocytes and endothelial cells, and promote the release of inflammatory mediators such as TNF. LBP can also function as an opsonin, promoting monocytes to engulf conditioned LPS and gram-negative bacteria; thus LBP can regulate inflammatory response induced by LPS [10]. The possible mechanism of Paeoniae Radix Alba in treating mastitis might be that paeoniflorin inhibits the expression of LBP and antagonize LBP-mediated LPS inflammatory response.

#### 3.3.4. Lactotransferrin

Lactotransferrin is a natural glycoprotein with immune functions that exists in breast milk. Its physiological functions include iron absorption promotion, immunomodulation, antibacterial, and antiviral effects,* etc.* [11]. The mechanism of Glycyrrhizae Radix et Rhizoma in treating mastitis might be that the glycine it contains can regulate lactotransferrin level, enhance the body's immunity and exert anti-inflammatory effects.

### 3.4. Results of Searching in BATMAN-TCM

#### 3.4.1. Key TCM Combination-Target-Disease Analysis

BATMAN-TCM was employed to perform molecule-target-disease analysis of the 12 key CMM combinations. Diseases related to mammary gland and mastitis were screened out. Results show that each key CMM combination is related to two to four of the diseases including inflammation, inflammatory disease, breast cancer, and hormone-dependent breast cancer (Table 6). Hormone-dependent breast cancer refers to that when tumor cells show positive expression of estrogen receptor (ER)/progesterone receptor (PR) and the growth and proliferation of tumor cells are regulated by estrogen and progesterone, antiestrogenic drugs must be used for treatment [12]. As can be seen, the key CMM combinations studied here are closely related to the treatment of mastitis and breast cancer.

#### 3.4.2. Key TCM Combination-Target-Signaling Pathway Analysis

BATMAN-TCM was used to perform signaling pathway enrichment analysis of the targets of 12 CMM combinations. Then the obtained signaling pathways were compared with those KEGG signaling pathways related to mastitis found in MalaCards. Four common signaling pathways were identified: NF-*κ*B signaling pathway, immune system, PI3K/Akt signaling pathway, and TNF signaling pathway. These four signaling pathways were the signaling pathways related to mastitis that CMMs involve in.

*(1) NF-κB Signaling Pathway*. Nuclear factor-*κ*B (NF-*κ*B) is a transcription factor widely existing in eukaryotic cells. NF-*κ*B normally exists in nonactivated state in cells. When cells are stimulated by stimulating factors such as inflammatory mediators, viral infection, oxidative stress,* etc.*, NF-*κ*B will be activated and transfer to cell nucleus. It will then bind to the enhancer sites of target genes such as cytokines, growth factors, intercellular adhesion molecule, acute phase protein,* etc.* and enhance their transcription. Therefore, NF-*κ*B signaling pathway plays a key role in regulating immune response, inflammatory response, cell proliferation/differentiation/apoptosis,* etc*. If the activation of NF-*κ*B signaling pathway cannot be timely inhibited, various pathological responses may occur [13]. In recent years, the relationship between NF-*κ*B signaling pathway and human diseases has received more and more attention. Research has shown that many CMMs which have significant efficacy in treating NF-*κ*B-related diseases can inhibit the activity of NF-*κ*B. By analyzing the working mechanism of CMM at cellular and molecular levels, it is found that CMM contain some active ingredients, which can regulate the activity of NF-*κ*B at cellular or molecular levels and thus exert therapeutic effects. Among the CMM extracts that can significantly inhibit the activity of NF-*κ*B, many are glycosides, including flavonoids, nonflavonoid polyphenols, and other glycosides [14]. Therefore, the mechanism of key CMM combinations in treating mastitis may be that they inhibit the activity of NF-*κ*B signaling pathway, block the NF-*κ*B-mediated expression of various cytokines, and thus exert therapeutic effects on mastitis.

*(2) Immune System*. The inappropriate activation of NF-*κ*B signaling pathway can not only cause inflammatory response, but also decrease the body's immunity [15]. When the body's immunity is low, infection may become severer. Therefore, in terms of mastitis, regulation of immune system is also a mechanism of drug treatment. Many CMMs show effects of immune enhancement. For example, astragalus root, radix ginseng, and tangshen can replenish qi and strengthen body resistance. Baikal skullcap root and amur cork-tree can remove heat and eliminate toxicity. Since NF-*κ*B signaling pathway is closely related to immunomodulation, the key CMM combinations studied here may exert immunomodulatory effects by regulating NF-*κ*B signaling pathways and immune system signaling pathways.

*(3) PI3K/Akt Signaling Pathways*. Phosphoinositide 3-kinase (PI3K) and its downstream target Akt are important signaling molecules and key survival factors that control cell proliferation, apoptosis, and tumorigenesis [16]. Research has shown that the enhancement of PI3K/Akt signaling pathway is one of the causes of hormonal therapy resistance in breast cancer. The inhibitors of many molecules in this signaling pathway can inhibit the growth of breast cancer cells and induce the apoptosis of cancer cells; thus they are often used as important drugs for treating breast cancer [17]. In addition, PI3K/Akt may affect the expression of proinflammatory cytokines and participate in inflammatory response by regulating TLR4 and its downstream molecules in macrophage. The phosphorylation of Akt can promote the phosphorylation of inhibitory subunit alpha of NF-*κ*B (I*κ*B-*α*). In this way, I*κ*B-*α* is separated from NF-*κ*B, which is thus activated and enter cell nucleus. Then, it can induce the expression of many inflammatory factors such as IL-6, tumor necrosis factor-*α* (TNF-*α*),* etc.* and cause inflammatory response [18]. Many CMMs have regulatory effects on PI3K/Akt signaling pathway. For example, hyperoside can regulate PI3K/Akt signaling pathway, decrease the activity of TNF-*α* and IL-6, and mitigate inflammatory response [19]. Astragalus polysaccharide can significantly decrease the expression of *ρ*-Akt and PI3K in colonic mucosa and exert therapeutic effects on colitis [20]. The key CMM combinations studied here may reduce the expression of proinflammatory cytokines by regulating PI3K/Akt signaling pathways and then exert anti-inflammatory effects.

*(4) TNF Signaling Pathway*. The activation of NF-*κ*B signaling pathway can promote the release of proinflammatory factors such as TNF-*α* and IL-6. This can enhance inflammatory response and cause pro-/anti-inflammation imbalance, finally leading to further enhancement of inflammatory response and immune disorders [21]. Inducible transcription factors NF-*κ*B family are activated in response to various stimuli. The most characteristic inducers are TNF cytokine family [22]. TNF is a major mediator of apoptosis, inflammation, and immunity. The activation of TNF signaling pathway is related to a wide range of human diseases, including septicemia, diabetes, cancer, osteoporosis, multiple sclerosis, rheumatoid arthritis, and inflammatory bowel disease [21]. CMMs which can remove heat and promote blood circulation contain active ingredients, which can inhibit the secretion of TNF, decrease the activity of NF-*κ*B and block the development of inflammation. Red sage, Chinese angelica, moutan bark, peony root, sanqi,* etc.* are frequently used CMMs that can promote blood circulation. These CMMs not only can promote blood circulation and resolve stasis, but also have anti-inflammatory effects [23, 24]. The mechanism of key CMM combinations studied here in treating mastitis might be that they reduce the secretion of TNF by inhibiting TNF signaling pathway and block the development of inflammation.

## 4. Discussion

The treatment of mastitis in western medicine mainly adopts beta-lactam antibiotics to sterilize and prevent infection. The mechanism of drug action is to kill bacteria by destroying the cell wall. In comparison, there are many kinds of Chinese materia medica for treating mastitis, and the efficacy of CMM prescriptions also includes clearing heat and detoxification, removing swelling from breast, soothing liver and regulating qi, etc. Therefore, the study on the mechanism of action of CMM prescriptions in the treatment of mastitis is more complicated. Thus there are few related reports, most of which are about the mechanism of action of single Chinese materia medica. Gao Ruifeng [25] reported that one of the main components of honeysuckle, chlorogenic acid, acts as an antimastitis mechanism by inhibiting the activation of TLR4 and NF-*κ*B signaling pathways. In addition, it can bind and activate PPAR- *γ* so that TLR4 can downregulate expression and inhibit the activation of downstream NF-*κ*B signaling pathway. Finally, the expression levels of genes and proteins of inflammatory factors such as TNF-*α*, IL-1*β*, and IL-6 were decreased. Zhao Yongwang [26] reported that the main ingredient of scutellaria baicalensis can stabilize the mast cell membrane and inhibit its degranulation to reduce the release of inflammatory mediators. In addition, it can regulate the secretion of TNF-*α* and IFN-*γ* compounds to maintain a certain level. It can not only participate in antibacterial immunity and prevent excessive inflammation of tissues, but also regulate cellular immunity and improve breast immunity.

In this paper, data mining method was used to statistically analyze the frequencies of CMMs in prescriptions for treating mastitis and find association rules. Key CMM combinations for treating mastitis were obtained and can provide useful information for clinical therapy of mastitis. Network pharmacology was employed to obtain the potential targets of high-frequency CMMs and the potential signaling pathways that key CMM combinations involve in. This provides a new method for the research of the mechanism of CMMs in treating mastitis. However, the results are only based on already-known chemical composition of CMMs, related targets, and signaling pathways. With the development of technology, new ingredients and targets will be found in CMMs and there will also be more disease-related information. This will help enrich the results of this paper. In addition, according to CMM combination-target-disease analysis results, many CMMs for treating mastitis are also related to the treatment of breast cancer. Therefore, our future research will focus on the difference between CMMs used for treating mastitis and breast cancer.

Due to the large number of ingredients in CMMs and the complicated interaction between CMM and human body, it remains difficult to elaborate the working mechanism of CMM. In fact, figuring out the working mechanism of CMM has become a bottleneck in the modernization and internationalization of CMM. From the perspective of network pharmacology, medicine-target-disease analysis may provide an entry point and a new strategy for further investigation into the working mechanism of CMM prescriptions.

## Figures and Tables

**Table 1:** All CMMs in DPTM and their frequencies (Fre).

CMM	Fre	CMM	Fre	CMM	Fre	CMM	Fre
Taraxaci Herba	99	Scutellaria barbata D. Don	16	semen euryalis ferocis	4	Saposhnikoviae Radix	1
Glycyrrhizae Radix et Rhizoma	98	Dendranthema indicum	15	caulis akebiae	4	rhizoma phragmitis communis	1
Paeoniae Radix Alba	64	Asarum sagittarioides C. F. Liang.	15	radix notoginseng	4	Cluster Mallow Fruit Fructus Malvae	1
semen citri reticulatae	60	Penthorum chinense Pursh	12	semen sinapis albae	4	Schizonepetae Herba	1
Bupleuri Radix	59	Forsythia suspensa	10	Ranunculus ternatus	4	rhizoma sparganii	1
pericarpium citri reticulatae viride	53	Fructus Camptothecae Acuminatae	10	nidis vespae	4	Fructus Toosendan	1
pericarpium trichosanthis kirilowii et multilobae	48	Trichosanthes kirilowii Maxim.	10	Carthamus tinctorius L.	3	semen pruni persicae	1
Semen Coicis	47	Fructus hordei Germinatus	10	caulis milletiae seu longae	3	Cuscuta japonica Choisy	1
herba houttuyniae	45	Gardenia jasminoides Ellis	9	Mahonia fortunei *（*Lindl. *）*Fedde	3	semen litchi chinensis	1
Semen Vaccariae	42	Manis pentadactyla	9	Anemarrhena asphodeloides Bunge	3	Amomi Fructus	1
Salvia miltiorrhiza Bunge	41	Prunella vulgaris L	9	fructus rosae laevigatae	3	cacumen bioyae orientalis	1
Bulbus Fritillariae Thunbergii	36	Platycodon grandiflorus	9	Manyinflorescenced sweetvetch root	3	Draconis Sanguis	1
Lamiophlomis rotata Kudo	35	Atractylodes Lancea (Thunb.) DC.	8	Herba Thlaspis	3	Hematite Haematitum	1
Astragalus membranaceus	33	Ligusticum chuanxiong hort	8	Massa Fermentata	3	Rhizoma smilacis glabrae	1
Poria	31	fructus liguidambaris taiwaniae	7	Leonurus Linn.	3	Glabrous greenbrier rhizome	1
Retinervus luffae Fructus	30	Bombyx Batryticatus	6	Codonopsis Radix	3	Corydalis yanhusuo W.T.Wang	1
Vigna angularis	30	radix paeoniae rubra	6	Fritillaria cirrhosa D. Don	2	Scrophularia ningpoensis Hemsl	1
Viola yedoensis Makino	30	thallus algae	6	Rehmannia glutinosa (Gaetn.) Libosch. ex Fisch. et Mey.	2	AIpiniatonkinensis Gagnep	1
Trichosanthis Radix	29	Rehmanniae Radix	6	Curcumae Radix	2	Notopterygium incisum Ting ex H. T. Chang	1
Sarcandraglabra	25	Alismatis Rhizoma	6	Rhizoma Pinelliae	2	Phellodendron amurense Rupr	1
Angelicae Sinensis Radix	25	Rhizoma Cyperi	6	Cornu Cervi Degelatinatum	2	Kadsura longipedunculata Finet et Gagnep	1
Curcumae Rhizoma	24	Sargassum	6	Itoa orientalis	2	Dendranthema morifolium*（*Ramat. *）*Tzvel	1
Zea mays L	24	Rhodiola rosea L.	6	Polygoni Multiflori Radix	2	Rubia cordifolia L	1
Atractylodes macrocephala Koidz	24	Radix Actinidiae Chinensis	6	Semen Juglandis	2	Curcumae Longae Rhizoma	1
Lonicera japonica Thunb	22	Hemsleya amabilis Diels	6	Hedyotis diffusa	2	Ginseng Radix et Rhizoma	1
Pericarpium Citri Reticulatae	21	Stemmacanthauniflora (L. ) Dittrich	5	semen cannabis sativae	2	Radix Aucklandiae	1
Scutellaria baicalensis Georgi	21	Rhizoma Paridis	5	Solanum nigrum Linn	2	radix linderae strychnifoliae	1
Crataegus pinnatifida Bunge	20	Arctium lappa L.	5	Rhei Radix et Rhizoma	2	radix angelicae pubescentis	1
Tetrapanax papyriferus	17	Myrrh	5	Folium Perillae	2	rhizoma arisaematis	1
Spina Gleditsiae	17	Gentiana scabra Bunge	5	Amygdalus Communis Vas	2	fructus germinatus oryzae sativae	1
ostrea gigas tnunb	17	Boswellia sacra	5	Gypsum Fibrosum	2	Arc Shell Concha Arcae	1
Angelicae Dahuricae Radix	16	fructus citri aurantii	4	cortex albizziae julibrissinis	1	Zingiber officinale Roscoe	1
Corni Cervi	16	Plantago asiatica L.	4	Semen Cassiae	1		

**Table 2:** Key CMM combinations in DPTM.

Number	Key CMM combinations
1	Taraxaci herba, Salvia miltiorrhiza Bunge, Paeoniae Radix Alba and Glycyrrhizae Radix et Rhizoma
2	Taraxaci herba, semen citri reticulatae, Paeoniae Radix Alba and Glycyrrhizae Radix et Rhizoma
3	Taraxaci herba, herba houttuyniae and Glycyrrhizae Radix et Rhizoma
4	Taraxaci herba, Semen Coicis and Vigna angularis
5	Taraxaci herba, herba houttuyniae and Semen Coicis
6	Taraxaci herba,herba houttuyniae and pericarpium trichosanthis kirilowii et multilobae
7	Taraxaci herba, trichosanthes kirilowii peel and Semen Coicis
8	Paeoniae Radix Alba, herba houttuyniae and Glycyrrhizae Radix et Rhizoma
9	Paeoniae Radix Alba, Bupleuri Radix and Glycyrrhizae Radix et Rhizoma
10	semen citri reticulatae, pericarpium citri reticulatae viride and Bupleuri Radix
11	Taraxaci herba and Viola philippica
12	Taraxaci herba and Lamiophlomis rotata Kudo

**Table 3:** Targets related to mastitis disease.

**Symbol**	**Description**	**Score**
LTF	Lactotransferrin	27.2
CXCL8	C-X-C Motif Chemokine Ligand 8	23.43
TLR2	Toll Like Receptor 2	23.13
IL6	Interleukin 6	22.28
LBP	Lipopolysaccharide Binding Protein	22.18
ALB	Albumin	22.16
CCL5	C-C Motif Chemokine Ligand 5	21.49
NOD2	Nucleotide Binding Oligomerization Domain Containing 2	21.14
IL17A	Interleukin 17A	20.81
HP	Haptoglobin	20.74
CP	Ceruloplasmin	20.69
CSF2	Colony Stimulating Factor 2	20.01
ICAM1	Intercellular Adhesion Molecule 1	19.99
CSN2	Casein Beta	13.66
LALBA	Lactalbumin Alpha	12.71
OXT	Oxytocin/Neurophysin I Prepropeptide	12.49
CXCL6	C-X-C Motif Chemokine Ligand 6	11.6
CSN3	Casein Kappa	11.3
SLPI	Secretory Leukocyte Peptidase Inhibitor	11.2
STAT5A	Signal Transducer And Activator Of Transcription 5A	10.48

**Table 4:** Signal pathways related to mastitis disease and top affiliating genes of pathways.

**Super pathways**	**Top Affiliating Genes**
Innate Immune System	CCL5,CSF2,CXCL8,HP,ICAM1,IL17A
Akt Signaling	CCL5,CSF2,CXCL6,CXCL8,IL17A,IL6
Cytokine Signaling in Immune system	CCL5,CSF2,CXCL8,ICAM1,IL17A,IL6
Toll-Like receptor Signaling Pathways	CCL5,CP,CXCL8,IL6,NOD2,TLR2
Influenza A	CCL5,CXCL8,ICAM1,IL6,TLR2
Kaposi's sarcoma-associated herpesvirus infection	CSF2,CXCL8,ICAM1,IL6,TLR2
Toll-like receptor signaling pathway	CCL5,CXCL8,IL6,LBP,TLR2
Selenium Micronutrient Network	ALB,CCL5,ICAM1,IL6
IL-17 Family Signaling Pathways	CSF2,CXCL6,CXCL8,IL17A,IL6,TLR2
Tuberculosis	IL6,LBP,NOD2,TLR2
Bacterial infections in CF airways	CXCL8,IL6,LBP,TLR2
Interleukin-4 and 13 signaling	CXCL8,ICAM1,IL17A,IL6,LBP
IL27-mediated signaling events	IL17A,IL6,TLR2
TNF signaling pathway	CCL5,CSF2,ICAM1,IL6,NOD2
AGE-RAGE signaling pathway in diabetic complications	CXCL8,ICAM1,IL6
Amoebiasis	CSF2,CXCL8,IL6,TLR2
NF-kappa B signaling pathway	CXCL8,ICAM1,LBP
Th17 Differentiation Pathway	IL17A,IL6,TLR2
Salmonella infection	CSF2,CXCL8,IL6,LBP
Pertussis	CXCL6,CXCL8,IL6
IgA-Producing B Cells in the Intestine	ICAM1,IL6,TLR2
Lung fibrosis	CCL5,CSF2,CXCL8,IL6
Photodynamic therapy-induced NF-kB survival signaling	CSF2,CXCL8,ICAM1,IL6
Glucocorticoid receptor regulatory network	CSF2,CSN2,CXCL8,ICAM1,IL6
Legionellosis	CXCL8,IL6,TLR2
Cytokine production by Th17 cells in CF	CSF2,CXCL6,CXCL8,ICAM1,IL17A,IL6
Malaria	CXCL8,ICAM1,IL6,TLR2
amb2 Integrin signaling	HP,ICAM1,IL6
Interleukin-10 signaling	CCL5,CSF2,CXCL8,ICAM1,IL6
Rheumatoid arthritis	CCL5,CSF2,CXCL6,CXCL8,ICAM1,IL17A
G-protein signaling_RhoB regulation pathway	CCL5,CSF2,CXCL6,CXCL8,IL17A,IL6
G-protein signaling_Rap2B regulation pathway	CCL5,CSF2,CXCL6,CXCL8,IL17A,IL6

**Table 5:** High correlation target of high frequency Chinese medicine in the treatment of mastitis diseases.

CMM	pharmaceutical molecule	Target
pericarpium citri reticulatae viride	hesperidin	ICAM-1
Paeoniae Radix Alba	paeoniflorin	IL-6
	
Bupleuri Radix	methyl palmitate, lauric acid	IL-6
Salvia miltiorrhiza Bunge	oleanolic acid, ursolic acid, luteolin, Tanshinone I, apigenin	ICAM-1
	glycine	lactotransferrin
Radix Salviae miltiorrhizae	glycyrrhizic acid	IL-6
Semen Vaccariae	quercetin	IL-6
herba houttuyniae	Caryophyllene,rutin,quercetin	IL-6
	Kaempferol, quercetin	ICAM-1
pericarpium trichosanthis kirilowii et multilobae	lauric acid, methyl palmitate	IL-6
	glycine	Lactotransferrin

**Table 6:** “Drug combination-target-disease” related to “breast” and “mastitis”.

No	key CMM combination	Target related diseases	Drug target
1	Taraxaci herba, semen citri reticulatae, Paeoniae Radix Alba and Glycyrrhizae Radix et Rhizoma	Inflammation	ADORA1;ADORA2A;PLA2G1B;PLD1;
		Breast Cancer	CYP19A1;ESR1;PGR;VDR;
		Inflammatory Diseases	ADORA1;CNR1;
		Breast Cancer (Hormone-Sensitive)	HSD17B1;
2	Taraxaci herba, Salvia miltiorrhiza Bunge, Paeoniae Radix Alba and Glycyrrhizae Radix et Rhizoma	Breast Cancer	CYP19A1;ESR1;PGR;VDR;
		Inflammation	ADORA1;ADORA2A;PLA2G1B;PLD1;
		Inflammatory Diseases	PIK3CD;
		Breast Cancer (Hormone-Sensitive)	HSD17B1;
3	Taraxaci herba, Radix Salviae miltiorrhizae, Paeoniae Radix Alba and Glycyrrhizae Radix et Rhizoma	Inflammation	ADK;ADORA1;ADORA2A;PLA2G1B;PLD1;PTGS2;
		Breast Cancer	CYP19A1;ESR1;PGR;PTGS2;VDR;
		Breast Cancer (Hormone-Sensitive)	HSD17B1;
4	Taraxaci herba, Semen Coicis and Vigna angularis	Breast cancer	ESR1;PGR;VDR;
		Inflammation	PLD1;
5	Taraxaci herba, Semen Coicis and Vigna angularis	Inflammation	ADK;PLA2G1B;PLD1;PTGS2;
		Inflammatory diseases	PTGS2;
		Breast cancer	ESR1;PGR;PTGS2;VDR;
6	Taraxaci herba,herba houttuyniae and pericarpium trichosanthis kirilowii et multilobae	Inflammation	ADK;ADORA1;PLA2G1B;PLD1;PTGS2;
		Breast cancer	ESR1;PGR;PTGS2;VDR;
		Inflammatory diseases	PTGS2;
7	Taraxaci herba, trichosanthes kirilowii peel and Semen Coicis	Inflammation	ADORA1;PLD1;
		Breast cancer	ESR1;PGR;VDR;
8	Taraxaci herba, trichosanthes kirilowii peel and Semen Coicis	Inflammation	ADK;ADORA1;ADORA2A;PLA2G1B;PTGS2;
		Breast cancer	CYP19A1;ESR1;PGR;PTGS2;VDR;
		Breast cancer (hormone-sensitive)	HSD17B1;
		Inflammatory diseases	PIK3CD;PTGS2;
9	Paeoniae Radix Alba, Bupleuri Radix and Glycyrrhizae Radix et Rhizoma	Inflammation	ADORA1;ADORA2A;PLA2G1B;PPARD;PPARG;PTGS2;
		Breast cancer	AKT1;CYP19A1;ESR1;PGR;PTGS2;VDR;
		Breast cancer (hormone-sensitive)	HSD17B1;
		Inflammatory diseases	PIK3CD;PTGS2;
10	semen citri reticulatae, pericarpium citri reticulatae viride and Bupleuri Radix	Inflammation	ADORA1;ADORA2A;PLA2G1B;PPARD;PPARG;PTGS2;
		Breast cancer	AKT1;CYP19A1;ESR1;PGR;PTGS2;VDR;
		Inflammatory diseases	PIK3CD;PTGS2;
11	Taraxaci herba and Viola philippica	Breast cancer	ESR1;PGR;VDR;
		Inflammation	PLA2G1B;PLD1;
12	Taraxaci herba and Lamiophlomis rotata Kudo	Breast cancer	ESR1;PGR;VDR;
		Inflammation	PLD1;

## Data Availability

The data that support the findings of this study are openly available in https://www.malacards.org/pages/info, http://lsp.nwu.edu.cn/tcmsp.php, http://bidd.nus.edu.sg/group/tcmsite/default.aspx, and http://bionet.ncpsb.org/batman-tcm/.
